# Supplementary material for: Trends in alcohol consumption in middle-aged and older adults, assessed with self-report and the alcohol marker phosphatidylethanol – A longitudinal HUNT study
Source: PLoS One. 2025 Oct 24;20(10):e0334556. doi: 10.1371/journal.pone.0334556 (PMC12551891; doi:10.1371/journal.pone.0334556)
Supplement: S1 Table — (DOCX) [file pone.0334556.s001.docx]

**S1 Table. Changes in questions used to define abstinence, current drinking, and heavy episodic drinking from HUNT2 (1995-1997) to HUNT3 (2006-2008) and HUNT4 (2017-2019)**

| **Drinking pattern** | **Questions in HUNT2 1995-97** | **Questions in HUNT3 2006-08** | **Questions in HUNT4 2017-19** |
| --- | --- | --- | --- |
| Abstinence | Q1: Concerning alcohol: Do you entirely abstain from alcohol (**yes^1^** or no) | Q1: About how often in the last 12 months did you drink alcohol? (do not include low-alcohol beer)  4-7 times a week  2-3 times a week  About once a week  2-3 times a month  About once a month  A few times a year  **Not at all the last year^1^**  **Never consumed alcohol^1^** | Q1: About how often in the last 12 months did you drink alcohol (do not include low-alcohol beer)  **Not at all last 12 months^1^**  Once a month or less  2-4 times a month  2-3 times a week  4 or more times a week  **I have never consumed alcohol^1^** |
| Current drinking | Q1: How many times a month do you normally drink alcohol? (Number of times___) (**Those reporting drinking once a month or more**)**^2^** | Q1: About how often in the last 12 months did you drink alcohol? (do not include low-alcohol beer)  4-7 times a week  2-3 times a week  About once a week  2-3 times a month  **About once a month^2^**  **A few times a year^2^**  Not at all the last year  Never consumed alcohol | Q1: About how often in the last 12 months did you drink alcohol (do not include low-alcohol beer)  Not at all last 12 months  **Once a month or less^2^**  2-4 times a month  2-3 times a week  4 or more times a week  I have never consumed alcohol |
| Heavy episodic drinking | No information | Q1: How often do you drink **5** glasses or more of beer, wine or spirits in one sitting?  Never  **Monthly^3^**  **Weekly^3^**  **Daily^3^** | Q1: How often do you drink **6** glasses or more of beer, wine or spirits in one sitting?  Never  Less than monthly  **Monthly^3^**  **Weekly^3^**  **Daily^3^** |

Abbreviations: HUNT = The Trøndelag Health Study; Q = questionnaire

^1^Response to the question used to define abstinence in HUNT2, HUNT3, and HUNT4

^2^Response to the question used to define current drinking in HUNT2, HUNT3, and HUNT4

^3^Response to the question used to define heavy episodic drinking in HUNT3 and HUNT4
